# Supplementary material for: Vaccine effectiveness of CoronaVac against COVID-19 among children in Brazil during the Omicron period
Source: Nat Commun. 2022 Aug 13;13:4756. doi: 10.1038/s41467-022-32524-5 (PMC9375192; doi:10.1038/s41467-022-32524-5)
Supplement: Supplementary file 1 — Supplementary Information [file 41467_2022_32524_MOESM1_ESM.pdf]

Supplementary Information

Vaccine effectiveness of CoronaVac against COVID-19 among children in Brazil during the Omicron period

Pilar T V Florentino*[1,2], Flávia J O Alves[1], Thiago Cerqueira-Silva[3,4], Vinicius de Araújo Oliveira[1,4], Juracy B S Júnior[5], Adelson G Jantsch[3], Gerson O. Penna[6], Viviane Boaventura[3,4], Guilherme L Werneck[7,8], Laura C Rodrigues[9], Neil Pearce[9], Manoel Barral-Netto[1,4], Mauricio L Barreto†[1], Enny S Paixão†[9]

† These authors jointly supervised this work
* Corresponding Author: pilar.veras@gmail.com

Affiliations

[1] Centre of Data and Knowledge Integration for Health (CIDACS), Instituto Gonçalo Moniz Institute, Oswaldo Cruz Foundation (Fiocruz), Salvador, Brazil
[2] Biomedical Science Institute, University of São Paulo, São Paulo, Brazil
[3] Gonçalo Moniz Institute, Oswaldo Cruz Foundation (Fiocruz), Salvador, Brazil
[4] Faculty of Medicine, Federal University of Bahia, Salvador, Brazil
[5] Public Health Institute, Federal University of Bahia, Salvador, Brazil
[6] Tropical Medicine Centre, University of Brasília, Fiocruz School of Government, Brasília, Brazil
[7] Department of Epidemiology, Social Medicine Institute, State University of Rio de Janeiro, Rio de Janeiro, Brazil.
[8] Institute of Collective Health Studies, Federal University of Rio de Janeiro, Rio de Janeiro, Brazil
[9] London School of Hygiene and Tropical Medicine, London, UK

Contents

TABLE S1: DEMOGRAPHIC FOR CHILDREN POPULATION FROM 6-11 YEARS OLD 2

TABLE S2. ICU ADMISSION CORONAVAC EFFECTIVENESS AMONG CHILDREN BETWEEN 6-11 YEARS ESTIMATED WITH FIRTH BIAS METHOD FOR RARE EVENTS.... 3

TABLE S3. CORONAVAC EFFECTIVENESS AMONG CHILDREN BETWEEN 6-11 YEARS BASED ON MULTIPLE IMPUTATION ANALYSIS OF MISSING VALUES RELATING TO ETHNICITY WHICH CORRESPONDS TO 19.4% FROM TOTAL SAMPLE. 3

TABLE S4. CORONAVAC EFFECTIVENESS FOR SYMPTOMATIC INFECTION AND HOSPITAL ADMISSION AMONG CHILDREN BETWEEN 6-11 YEARS WITHOUT PREVIOUS INFECTION. 3

TABLE S5. CHECKLIST: THE RECORD STATEMENT[1]. 4

FIGURE S1 10

FIGURE S2 11

SUPPLEMENTARY NOTE 1: DETAILED INFORMATION OF BRAZILIAN DATABASES.... 11

REFERENCES.. 13

Table S1: Demographic for children population from 6-11 years old

Characteristic	Cases n = 89,595	Controls n = 108,363
**Age**		
6	12,540 (14.0%)	19,334 (17.8%)
7	13,865 (15.5%)	19,071 (17.6%)
8	14,785 (16.5%)	18,227 (16.8%)
9	15,475 (17.3%)	18,028 (16.6%)
10	16,277 (18.2%)	17,905 (16.5%)
11	16,653 (18.6%)	15,798 (14.6%)
**Sex**		
Female	42,475 (47.4%)	53,145 (49.0%)
Male	47,120 (52.6%)	55,218 (51.0%)
**Ethnicity**		
White	43,834 (48.9%)	51,086 (47.1%)
Black	1,694 (1.9%)	2,924 (2.7%)
Asian	813 (0.9%)	969 (0.9%)
Mixed	24,753 (27.6%)	33,247 (30.7%)
Indegenous	146 (0.2%)	193 (0.2%)
Missing	18,355 (20.5%)	19,944 (18.4%)
**Deprivation Index (quintile)**		
1 (least)	25,263 (28.2%)	44,076 (40.7%)
2	16,929 (18.9%)	19,115 (17.6%)
3	18,360 (20.5%)	18,490 (17.1%)
4	17,922 (20.0%)	16,271 (15.0%)
5 (most)	11,121 (12.4%)	10,411 (9.6%)
**Region of residence**		
Central West	9,371 (10.5%)	8,919 (8.2%)
North	4,473 (5.0%)	4,568 (4.2%)
Northeast	11,921 (13.3%)	12,648 (11.7%)
South	21,996 (24.6%)	24,488 (22.6%)
Southeast	41,834 (46.7%)	57,740 (53.3%)
**Number of comorbiditiies**		
0	87,136 (97.3%)	104,000 (96.0%)
1	2,381 (2.7%)	4,259 (3.9%)
>=2	78 (0.1%)	104 (0.1%)
**Previous confirmed infection**		
No	88,386 (98.7%)	105,592 (97.4%)
6-3 month	118 (0.1%)	330 (0.3%)
>6 month	1,091 (1.2%)	2,441 (2.3%)
**Hospital admission**		
No event	89087 (99.4%)	107123 (98.9%)
Yes	508 (0.6%)	1,240 (1.1%)
**Death**		
No event	89,556 (100%)	108,339 (100%)
Yes	39 (0.0%)	24 (0.0%)

Table S2. ICU admission CoronaVac effectiveness among children between 6-11 years estimated with Firth bias method for rare events.

ICU admission					
**Vaccination Status**	**positive tests n = 108**	**negative tests n = 108,363**	**OR CRUDE (95% CI)**	**OR ADJUSTED (95% CI)**	**VE (%) (95% CI)**
Unvaccinated	88 (0.13%)	69,923 (99.87%)			
**1st dose**					
0-13 days	7 (0.10%)	6,862 (99.90%)	0.81 (0.38,1.75)	0.80 (0.34, 1.61)	20.2 (-61.3, 65.9)
≥14-2nd dose	10 (0.05%)	20,193 (99.95%)	0.39 (0.20,0.76)	0.58 (0.28, 1.10)	41.9 (-10.4, 72.2)
**2nd dose**					
0-13 days	1 (0.02%)	4,552 (99.98%)	0.17 (0.02, 1.25)	0.62 (0.07, 2.48)	37.8 (-147.7, 93.2)
≥14 days	2 (0.03%)	6,833 (99.97%)	0.23 (0.06, 0.94)	0.79 (0.15, 2.77)	20.9 (-177.2, 85.0)

Table S3. CoronaVac effectiveness among children between 6-11 years based on multiple imputation analysis of missing values relating to ethnicity which corresponds to 19.4% from total sample.

**Multiple Imputation**	**VE (%) (95% CI)**	
**Vaccination Status**	**Symptomatic Infection**	**Severe Outcomes**
Unvaccinated		
**1st dose**		
0-13 days	[-6.6 (-10.7,-2.70]	21.8 (-13.4,46.0)
≥14-2nd dose	23.3 (20.7,25.8)	43.6 (21.4,59.5)
**2nd dose**		
0-13 days	32.9 (26.4,38.7)	81.8 (25.8,95.5)
≥14 days	42.5 (36.6,47.8)	56.7 (-2.1,81.6)

Table S4. CoronaVac effectiveness for symptomatic infection and hospital admission among children between 6-11 years without previous infection.

				**VE% (95% CI)**	
**Vaccination Status**	**Negative tests**	**Positive tests Infection**	**Positive tests Hospital admission**	**Symptomatic Infection**	**Hospital admission**
Unvaccinated	68,226	71,703 (51.24%)	434 (0.63%)		
**1st dose**					
0-13 days	6,699	7,419 (52.55%)	32 (0.48%)	[-97 (-13.9,-5.6)]	22.4 (-10.7,47.4)
≥14-2nd dose	19,657	8,136 (29.27%)	44 (0.22%)	20.4 (17.7,23.0)	44.1 (23.0,60.4)
**2nd dose**					
0-13 days	4,402	620 (12.35%)	2 (0.05%)	30.3 (23.6,36.5)	82.1 (43.0,97.1)
≥14 days	6,608	508 (7.14%)	6 (0.09%)	40.3 (34.2,45.9)	58.0 (8.6, 84.0)

Table S5. Checklist: The RECORD statement[1].

	Item No.	STROBE items	RECORD items	Location in manuscript where items are reported
	1	(a) Indicate the study's design with a commonly used term in the title or the abstract (b) Provide in the abstract an informative and balanced summary of what was done and what was found	RECORD 1.1: The type of data used should be specified in the title or abstract. When possible, the name of the databases used should be included.<br><br>RECORD 1.2: If applicable, the geographic region and timeframe within which the study took place should be reported in the title or abstract.<br><br>RECORD 1.3: If linkage between databases was conducted for the study, this should be clearly stated in the title or abstract.	Title: *"Vaccine effectiveness of CoronaVac against symptomatic and severe COVID-19 among children in Brazil during the Omicron period ".*<br><br>The abstract has the requested information
Background rationale	2	Explain the scientific background and rationale for the investigation being reported		Paragraph 1
Objectives	3	State specific objectives, including any prespecified hypotheses		Paragraph 2
Study Design	4	Present key elements of study design early in the paper		Paragraph 1 of the Methods section.
Setting	5	Describe the setting, locations, and relevant dates, including periods of recruitment, exposure, follow-up, and data collection		Paragraph 1 and 2 of the Methods section, including references that describe The statistical analysis plan (SAP).
Participants	6	*(a) Cohort study* - Give the eligibility criteria, and the sources and methods of selection of participants. Describe methods of follow-up *Case-control study* - Give the eligibility	RECORD 6.1: The methods of study population selection (such as codes or algorithms used to identify subjects) should be listed in detail. If this is not possible, an explanation should be provided.<br><br>RECORD 6.2: Any validation studies of the codes or algorithms used to select the population should be referenced. If validation was conducted for this study and not published elsewhere, detailed	The data source and linkage process are described in the methods section, including references that describe the linkage algorithm. Paragraphs 1,2 and 6 and Figure S1(Supplementary Material), including references that describe The statistical analysis plan (SAP).

		criteria, and the sources and methods of case ascertainment and control selection. Give the rationale for the choice of cases and controls<br>*Cross-sectional study* - Give the eligibility criteria, and the sources and methods of selection of participants<br><br>*(b) Cohort study* - For matched studies, give matching criteria and number of exposed and unexposed<br>*Case-control study* - For matched studies, give matching criteria and the number of controls per case	methods and results should be provided.<br><br>RECORD 6.3: If the study involved linkage of databases, consider use of a flow diagram or other graphical display to demonstrate the data linkage process, including the number of individuals with linked data at each stage.	
Variables	7	Clearly define all outcomes, exposures, predictors, potential confounders, and effect modifiers. Give diagnostic criteria, if applicable.	RECORD 7.1: A complete list of codes and algorithms used to classify exposures, outcomes, confounders, and effect modifiers should be provided. If these cannot be reported, an explanation should be provided.	Paragraph 3 and 4
Data sources/ measurement	8	For each variable of interest, give sources of data and details of methods of assessment (measurement). Describe comparability of assessment methods if there is more than one group		Paragraph 6
Bias	9	Describe any efforts to address potential sources of bias		Paragraph 1

Study size	10	Explain how the study size was arrived at		Figure S1(Supplementary Material)
Quantitative variables	11	Explain how quantitative variables were handled in the analyses. If applicable, describe which groupings were chosen, and why		Paragraph 3 and 4, and Table S1.
Statistical methods	12	(a) Describe all statistical methods, including those used to control for confounding<br>(b) Describe any methods used to examine subgroups and interactions<br>(c) Explain how missing data were addressed<br>(d) *Cohort study* - If applicable, explain how loss to follow-up was addressed<br>*Case-control study* - If applicable, explain how matching of cases and controls was addressed<br>*Cross-sectional study* - If applicable, describe analytical methods taking account of sampling strategy<br>(e) Describe any sensitivity analyses		Paragraph 1,2 and 5, including references that describe The statistical analysis plan (SAP).
Data access and cleaning methods		..	RECORD 12.1: Authors should describe the extent to which the investigators had access to the database population used to create the study population.<br><br>RECORD 12.2: Authors should provide information on the data cleaning methods used in the study.	Paragraph 6, including references that describe The statistical analysis plan (SAP) (paragraph 2).
Linkage		..	RECORD 12.3: State whether the study included person-level,	Paragraph 1,2 and 6, including references that

			institutional-level, or other data linkage across two or more databases. The methods of linkage and methods of linkage quality evaluation should be provided.	describe The statistical analysis plan (SAP).
Participants	13	(a) Report the numbers of individuals at each stage of the study (*e.g.*, numbers potentially eligible, examined for eligibility, confirmed eligible, included in the study, completing follow-up, and analysed)<br>(b) Give reasons for non-participation at each stage.<br>(c) Consider use of a flow diagram	RECORD 13.1: Describe in detail the selection of the persons included in the study (*i.e.,* study population selection) including filtering based on data quality, data availability and linkage. The selection of included persons can be described in the text and/or by means of the study flow diagram.	Paragraph 1 of the Result section.
Descriptive data	14	(a) Give characteristics of study participants (*e.g.*, demographic, clinical, social) and information on exposures and potential confounders<br>(b) Indicate the number of participants with missing data for each variable of interest<br>(c) *Cohort study* - summarise follow-up time (*e.g.*, average and total amount)		Paragraph 1
Outcome data	15	*Cohort study* - Report numbers of outcome events or summary measures over time<br>*Case-control study* - Report numbers in each exposure category, or		Tables S1, 1, Figures 1 and S1

		summary measures of exposure *Cross-sectional study* - Report numbers of outcome events or summary measures		
Main results	16	(a) Give unadjusted estimates and, if applicable, confounder-adjusted estimates and their precision (e.g., 95% confidence interval). Make clear which confounders were adjusted for and why they were included<br>(b) Report category boundaries when continuous variables were categorized<br>(c) If relevant, consider translating estimates of relative risk into absolute risk for a meaningful time period		Paragraph 1<br>Table 1, Figures S1
Other analyses	17	Report other analyses done—e.g., analyses of subgroups and interactions, and sensitivity analyses		Paragraph 1 and Supplemetary material
Key results	18	Summarise key results with reference to study objectives		Paragraph 1 of the discussion section.
Limitations	19	Discuss limitations of the study, taking into account sources of potential bias or imprecision. Discuss both direction and magnitude of any potential bias	RECORD 19.1: Discuss the implications of using data that were not created or collected to answer the specific research question(s). Include discussion of misclassification bias, unmeasured confounding, missing data, and changing eligibility over time, as they pertain to the study being reported.	Paragraph 2

Interpretation	20	Give a cautious overall interpretation of results considering objectives, limitations, multiplicity of analyses, results from similar studies, and other relevant evidence		Paragraph 3
Generalisability	21	Discuss the generalisability (external validity) of the study results		Paragraph 2 and 3
Funding	22	Give the source of funding and the role of the funders for the present study and, if applicable, for the original study on which the present article is based		We included a statement about the funding role.
Accessibility of protocol, raw data, and programming code		..	RECORD 22.1: Authors should provide information on how to access any supplemental information such as the study protocol, raw data, or programming code.	We included a section on data availability.

Figure S1

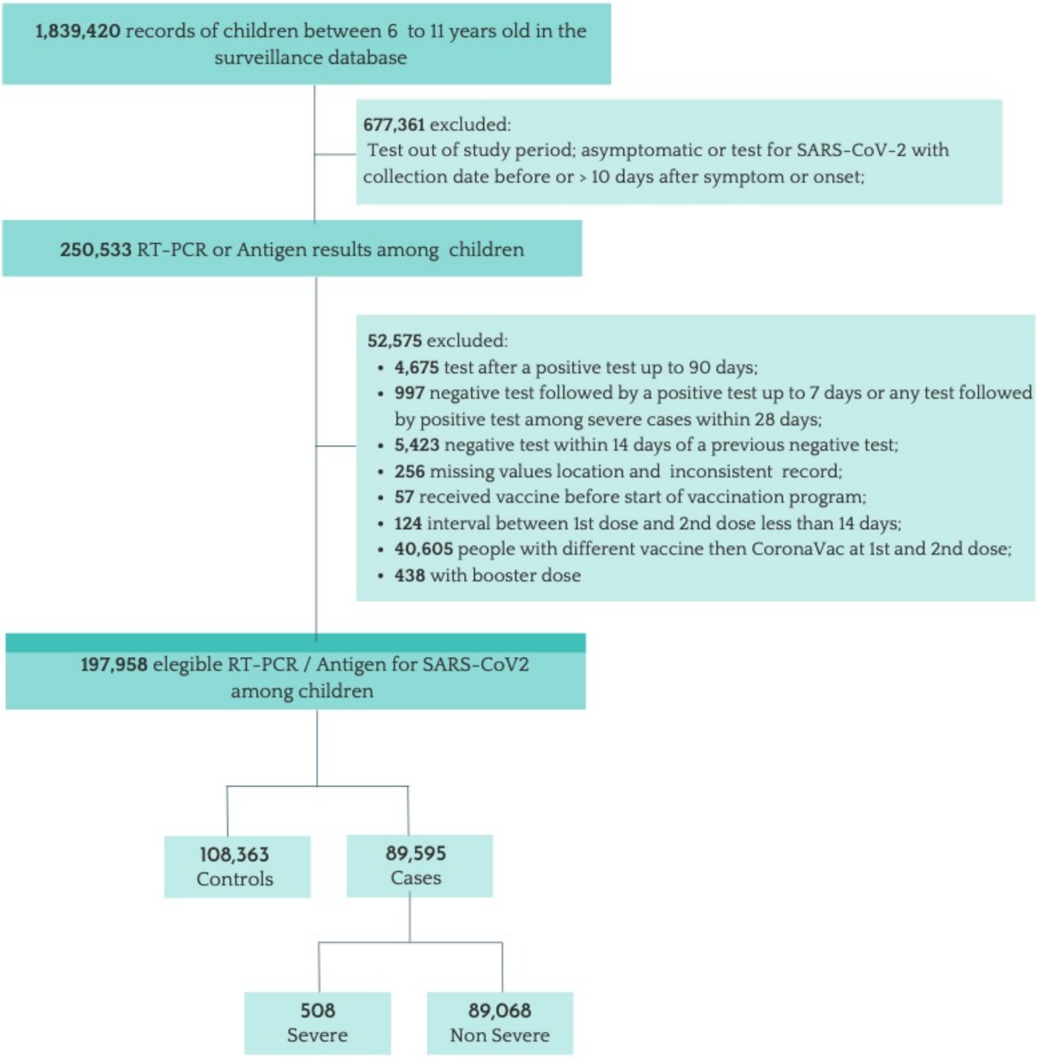

Figure S1. Flowchart of the selection of cases and controls among children between 6-11 years from surveillance databases. Individuals with an antigen or RT–PCR tests with sample collected within 10 of symptom onset were considered eligible.

Figure S2

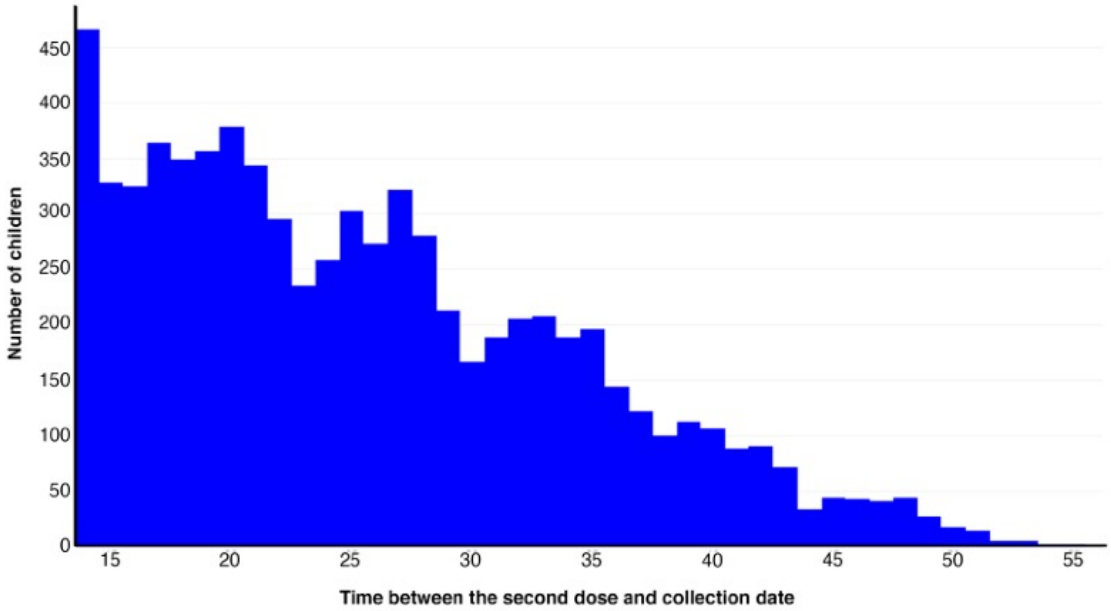

Figure S2. Distribution of tests among children between 6-11 years through time (in days) between second dose and collection date.

Supplementary Note 1: Detailed information of Brazilian Databases

The **e-SUS Notifica**: This database presents registration of all suspected cases of Covid-19 recorded in Brazil. This dataset includes all positive and negative RT-PCR test results from public and private healthcare system. Also, it contains the individual information on city of residence, demographic, and clinical data, such as the presence of comorbidities (Cardiac disease, Diabetes mellitus, Obesity, Immunosuppression, and chronic kidney disease), pregnancy status and set of symptoms. The acute respiratory diseases is defined as the presence of at least two of the following signs and symptoms: fever (even if referred), chills, sore throat, headache, cough, runny nose, loss or change to a sense of smell or taste.[2] Asymptomatic individuals or without register of symptoms were not included in this study, independent of the test result. This database has been used elsewhere as a data source for epidemiological research [3].

The **SIVEP-Gripe** is the Brazilian registration for severe acute respiratory syndrome (SARS, created after the Influenza pandemic of 2009. In 2020, it was expanded to include Covid-19. All Covid-19 hospitalisations from public and private healthcare are supposed to be

notified. In these registers, there are also individual information on city of residence, demographic, and clinical data, such as the presence of comorbidities (Cardiac disease, Diabetes mellitus, Obesity, Immunosuppression, and chronic kidney disease), pregnancy status and set of symptoms. In this system, SARS is defined as an individual who presents at least two mild symptoms) fever (even if referred), chills, sore throat, headache, cough, runny nose, loss or change to a sense of smell or taste) and dyspnea/respiratory discomfort, persistent pressure or pain in the chest, oxygen saturation less than 95% without oxygen, or cyanosis of the lips or face.[2] Also, for children the symptoms may include air loss, dehydration, or loss of appetite. Individuals who died with severe acute respiratory illness independent of hospitalisation are also registered. This database has also been widely used as a source for epidemiological studies.[4,5]

The **SI-PNI** contains data on all vaccines administered in Brazil. Covid-19 vaccines are administered by public health services and recorded in point-of-care applications. From SI-PNI, we extracted information on which Covid-19 vaccine was administered in first and second doses. By linking the vaccination data with the data on children from e-SUS Notifica and SIVEP-Gripe, we determined: (i) who tested negative for Covid-19 had been vaccinated (ii) with confirmed symptomatic Covid-19 infections had been vaccinated; and (iii) with SARS tested positive or negative for SARS-CoV-2. Children whose records from the other two databases described did not link to an SI-PNI vaccination record or if at the time of testing was not yet vaccinated were considered as unvaccinated.

References

1. Benchimol, E. I. *et al.* The REporting of studies Conducted using Observational Routinely-collected health Data (RECORD) Statement. *PLoS Medicine* **12**, 1–22 (2015).
2. Saiba como é feita a definição de casos suspeitos de Covid-19 no Brasil. *Ministério da Saúde* (2021). Available at: https://www.gov.br/saude/pt-br/coronavirus/artigos/definicao-e-casos-suspeitos. (Accessed: 12th May 2021)
3. Lima, F. E. T. *et al.* Time interval between onset of symptoms and COVID-19 testing in Brazilian state capitals, August 2020. *Epidemiologia e servicos de saude : revista do Sistema Unico de Saude do Brasil* **30**, e2020788 (2020).
4. Ranzani, O. T. *et al.* Characterisation of the first 250 000 hospital admissions for COVID-19 in Brazil: a retrospective analysis of nationwide data. *The Lancet Respiratory Medicine* **9**, 407–418 (2021).
5. Oliveira, E. A. *et al.* Clinical characteristics and risk factors for death among hospitalised children and adolescents with COVID-19 in Brazil: an analysis of a nationwide database. *The Lancet Child and Adolescent Health* **5**, 559–568 (2021).
